# Trauma subgroups among individuals with eating disorders: a latent profile analysis

**DOI:** 10.1186/s40337-026-01688-6

**Published:** 2026-07-10

**Authors:** Sinead Day, Nora Trompeter, Mandy Goldstein, Megan Bray, Phillipa Hay, Gabriella Heruc, Marion Roberts, Deborah Mitchison

**Affiliations:** 1 Graduate School of Health, University of Technology Sydney, Building 20, 100 Broadway, Ultimo, Sydney, NSW 2148 Australia; 2 University College London, London, UK; 3 everyBody Psychology & Wellbeing, 405/3 Waverley St, Bondi Junction, Sydney, NSW 2022 Australia; 4 School of Medicine, Western Sydney University, Locked Bag 1797, Penrith, NSW 2751 Australia; 5 Collective Health Co, 14/354 Mons Rd, Forest Glen, QLD 4556 Australia; 6 Translational Health Research Institute, School of Medicine, Western Sydney University, Locked Bag 1797, Penrith, Sydney, NSW 2751 Australia; 7 Mental Health Services Camden and Campbelltown Hospitals, South West Sydney Local Health District, Sydney, Australia; 8 Eating Disorders and Nutrition Research Group, Translational Health Research Institute, School of Medicine, Western Sydney University, Sydney, Australia; 9 Faculty of Medical and Health Sciences, Department of General Practice and Primary Healthcare, University of Auckland, 28 Park Ave, Grafton, Auckland, 1023 New Zealand; 10 Nurture Psychology, 204/100 Parnell Rd, Parnell, Auckland, 1052 New Zealand; 11 Graduate School of Health, University of Technology Sydney, Building 20, 100 Broadway, Ultimo, Sydney, NSW 2148 Australia

## Abstract

**Objective:**

Trauma-related symptoms are known to be highly prevalent in eating disorder (ED) populations but there has been limited research on complex post-traumatic stress disorder (CPTSD). The current study used latent profile analysis to examine trauma-related subgroups among clients with EDs and associations with ED severity and other outcomes.

**Methods:**

Participants (*N* = 378) attending residential, day program, and outpatient ED services completed measures of ED symptoms, body mass index (BMI), ED-related impairment, depression, anxiety, and trauma-related symptoms (based on ICD-11 PTSD and CPTSD criteria, and dissociation) at admission.

**Results:**

Latent profile analysis identified four trauma profiles: 1) high disturbances in self-organisation (DSO), 2) CPTSD (high on both PTSD and DSO symptoms, and severe dissociation), 3) general distress (moderate outcomes across all domains), and 4) no/mild trauma-related symptoms. After adjusting for level of care, profiles significantly differed on all outcomes except BMI, ED diagnosis, and objective binges. Groups 1 and 2 reported more severe ED and comorbid symptoms than groups 3 and 4, with group 2 also being more severe than group 1 on total ED psychopathology, eating concerns, purging, and anxiety.

**Discussion:**

Findings support previous research on the high prevalence of trauma-related symptoms in EDs, with DSO symptoms (unique to CPTSD) appearing to drive much of the association with ED symptom severity. ED services may benefit from targeted use of trauma-focused therapies for individuals presenting with high DSO symptoms, both in the presence and absence of severe PTSD symptomatology.

## Plain Language

Trauma-related symptoms are common among individuals with eating disorders but there has been limited research on specific types, including complex post-traumatic stress disorder (CPTSD). This study analysed patterns of trauma-related symptoms in people with eating disorders and how they were related to their eating disorder and other outcomes. The key patterns identified were: 1) people with high levels of symptoms unique to CPTSD (disturbances in self-organisation); 2) people with high levels of both these symptoms (disturbances in self-organisation), post-traumatic stress disorder (PTSD) symptoms, and dissociation; 3) people with moderate trauma-related symptoms of all kinds (general distress); and 4) no/mild trauma-related symptoms. The first and second groups had more severe eating disorder symptoms, mood and impairment. These findings support the importance of CPTSD symptoms in understanding associations between trauma and eating disorders.

## Introduction

Experiences of trauma such as childhood abuse and neglect are common among individuals with eating disorders (EDs), with an estimated 21–59% reporting childhood maltreatment in a meta-analysis by Molendijk et al. [35]. Consequently, those with EDs show high rates of comorbid trauma-related symptoms. Approximately one in four to one in two individuals with EDs meet criteria for post-traumatic stress disorder (PTSD), with higher rates seen in more intensive care settings [6, 17, 50, 51]. However, since the introduction of complex PTSD (CPTSD) to the ICD-11 World Health Organisation [WHO] [61], emerging findings suggest that the majority of cases of comorbid PTSD in EDs may be better captured by a CPTSD diagnosis. In the only study of CPTSD prevalence in EDs to date, Day, Hay, et al. [11, 12] found that approximately 28% of ED treatment-seekers met criteria for probable ICD-11 CPTSD and 4% for PTSD, with CPTSD being more common among residential and day program attendees than outpatient services. PTSD symptoms include intrusive thoughts/memories, avoidance of trauma-related cues, and heightened threat perception, which in CPTSD are present in addition to disturbances of self-organisation (DSO); namely, emotion dysregulation, relationship difficulties, and a core negative sense of self [61]. While these DSO symptoms present some similarities to non-trauma-related symptoms observed in other mental health presentations, emerging evidence supports them as distinctly trauma-related [20, 28].

While diagnosable trauma-related disorders are common among individuals with EDs, severe trauma-related symptoms that do not meet full diagnostic criteria appear even more prevalent. Day, Hay et al. [11, 12] found that 32% of participants with EDs showed severe PTSD symptoms and 58% reported severe DSO symptoms (at or exceeding the threshold required for a clinical diagnosis), with both symptom domains associated with more severe ED psychopathology. Similarly, severe levels of both PTSD and DSO symptoms—regardless of diagnostic status—have also each been found to moderate outcomes from ED residential care [13, 14]. As such, ongoing debate about PTSD and CPTSD diagnostic criteria belie a more complicated picture of trauma-related presentations in individuals with EDs that goes beyond diagnostic prevalence alone.

Given the range of potential trauma-related symptoms, further exploration of the ways in which they manifest in individuals with EDs is important in determining implications for treatment planning. For example, studies have documented that subclinical PTSD symptoms are associated with more severe impairment and depression (compared to those without PTSD symptoms) in first responders [10] and high DSO symptoms in the absence of PTSD symptoms are similarly associated with elevated depression and anxiety and lower functioning in general and veteran populations [18, 49, 54]. This also appears to be true for individuals in treatment for EDs [11–14]. What is not yet known is whether—outside of diagnosable cases of PTSD and CPTSD—trauma-related symptoms may cluster together in ways that predict variation in ED and associated psychopathology. Furthermore, trauma-related symptoms beyond PTSD and CPTSD have had limited investigation in ED populations. This is similarly true for dissociation, which is a response to trauma involving a disconnected experience of reality (including thoughts, emotions, memories, and identity), and commonly occurs within PTSD but is not a core diagnostic criterion [2, 32]. Rates of severe dissociation have been found to be elevated in individuals with EDs and associated with prior trauma exposure and poorer treatment outcomes [33, 40]; however, whether and in what ways dissociation clusters with PTSD and CPTSD symptoms in individuals with EDs remains unknown.

The aim of this study was therefore to investigate patterns of trauma-related symptoms in individuals with EDs and associations with ED symptoms, comorbid psychopathology, and functioning. This technique has been used in differentiating symptom profiles among trauma-related disorders [27], but has not, to our knowledge, been used to explore trauma-related latent groups in individuals with EDs using PTSD and CPTSD symptoms. Given the limited literature available particularly for CPTSD and dissociation in EDs, this study was exploratory in nature and no specific hypotheses were made.

## Method

### Participants

The sample consisted of 378 individuals presenting for eating disorder treatment at residential (*n* = 71), day program (*n* = 22), and outpatient (*n* = 285) levels of care, accessed through the TrEAT Registry (ACSQHC-ARCR-279) (Australian Commission on Safety and Quality in Health Care, n.d.; [53]). Outpatient services consisted of private practices across Australia and New Zealand in which individuals received individual therapy (typically 1-h weekly or fortnightly) with a psychologist. The residential service was Wandi Nerida, located in Queensland, Australia (mixture of public and private beds), and the day program was a public service located in Brisbane, Australia. Further description of these services is published elsewhere [12–14]. Eligible participants were 18 years or older, had a primary ED diagnosis, English language proficiency, and were willing and able to consent to the study. Ethics approval was granted by ethics committees at Western Sydney University (approval numbers H14742 and H14478) and the Royal Brisbane Hospital (EC00172).

### Measures

Participants reported demographic characteristics. ED diagnosis and body mass index (BMI) were reported by clinicians based on intake assessment. All measures were completed at admission to treatment.

### Eating disorder symptoms

The Eating Disorder Examination Questionnaire (EDE-Q v.6; [16]) measured ED psychopathology. Scores are averaged to provide a global index of ED psychopathology as well as four attitudinal subscales (restraint, eating concerns, weight concerns, shape concerns). Scores range from 0 to 6, with higher scores reflecting more severe symptoms. The EDE-Q also includes behavioural items in which individuals self-report the frequency of ED behaviours over the past 28 days. ED behaviours of objective and subjective binge episodes, purging (self-induced vomiting and laxative use), and compulsive exercise were examined in the current study. The EDE-Q has shown good validity and internal consistency in Australian community samples [36, 37], with acceptable internal consistency also found in the current sample for the global score (Cronbach’s α = 0.94) in addition to each subscale (α = 0.88 restraint, α = 0.72 eating concerns, α = 0.91 shape concerns, α = 0.85 weight concerns).

ED-related impairment was assessed with the Clinical Impairment Assessment (CIA; [3]). The CIA includes 16 Likert-type items (scored 0 to 3) that assess the impact of eating disorder symptoms on every day functioning across a variety of domains. Scores are summed to create a total score ranging from 0 to 48, with higher scores indicating greater impairment. This scale has shown acceptable reliability and a clinical cut-off score of 16 in previous research [46, 48]. The CIA showed good internal consistency in the current sample (Cronbach’s α = 0.95).

### Depression and anxiety

In the residential and day program samples, depression was measured using the Patient Health Questionnaire (PHQ-9; [29]) and anxiety was measured using the Generalised Anxiety Disorder Screener-7 (GAD-7; [52]). Scores on these scales range from 0–27 for depression and 0–21 for anxiety, with higher scores indicating more severe symptoms. Both scales have shown acceptable psychometric properties [9, 45]. In the outpatient group, depression and anxiety were measured using subscales of the Depression Anxiety Stress Scales-21 (DASS-21; [34]), which has similarly been widely validated [30, 44]. Depression and anxiety scores on the DASS range from 0 to 42 (higher scores represent more severe symptoms). All measures of anxiety and depression showed adequate internal consistency in the current sample (Cronbach’s α for PHQ-9 = 0.84, GAD-7 = 0.91, DASS-21 depression = 0.92, DASS-21 anxiety = 0.84).

### Trauma-related symptoms and disorders

PTSD and CPTSD symptoms were measured using the International Trauma Questionnaire (ITQ; [8]). This 18-item measure is derived from ICD-11 diagnostic criteria. The ITQ measures two domains of trauma-related symptoms, PTSD and DSO symptoms, each which also have three subscales: re-experiencing, avoidance, and sense of threat for PTSD symptoms; and affective dysregulation, negative self-concept, and disturbances in relationships for DSO symptoms. Scores on each domain and subscale can be summed to create a continuous index of severity ranging from 0 to 24 for domains (PTSD, DSO) and 0–8 for individual subscales, with higher scores indicating more severe trauma-related symptoms. The ITQ is also able to indicate probable PTSD and CPTSD diagnoses if specific thresholds are met for the symptom domains as well as functional impairment resulting from these symptoms. For a probable diagnosis of PTSD, participants must endorse at least one symptom from each PTSD subscale at ‘moderately’ or higher and report at least moderate resulting functional impairment. For a probable diagnosis of CPTSD, participants must endorse at least one symptom from each PTSD *and* DSO subscale at ‘moderately’ or higher, and report functional impairment from DSO symptoms. When criteria are met for probable CPTSD, no comorbid diagnosis of PTSD can also be made. The validity of the ITQ in distinguishing between PTSD and CPTSD under ICD-11 diagnostic criteria has been established in multiple studies [7, 38]. There was good internal consistency in the current sample for both PTSD (Cronbach’s α = 0.92) and CPTSD subscales (Cronbach’s α = 0.88).

As the ITQ does not include questions on dissociation, two items were adapted from the Global Psychotrauma Screen [43] to measure severity of dissociative symptoms: “I perceived or experienced the world or other people differently, so that things felt dreamlike, strange or unreal”, and “I felt detached or separated from my body”. Dissociation items were scored on the same Likert scale as the ITQ and summed to create a total score. They showed good internal consistency (Cronbach’s α = 0.85).

### Data analysis

Latent profile analysis was conducted using Stata Version 18 and further analyses were conducted using R Statistical Software (v4.3.1; [47]). Descriptive statistics were calculated including prevalence of trauma-related symptoms above the clinical threshold and rates of probable PTSD and CPTSD. Missing data ranged from 1.1 to 9.8% and was not associated with ED diagnosis, gender, level of care, or age (all *p* > 0.05 on Chi-square analyses and independent samples t-test).

Latent profile analysis was used to identify profiles of trauma-related symptoms. Latent class/profile analysis is a “bottom-up” statistical approach for revealing meaningful subgroups (as opposed to a top-down approach, such as in a diagnostic framework) based on shared patterns of symptoms and other characteristics [59, 60]. Seven indicator variables were included: the three PTSD symptom subscales (re-experiencing, avoidance, sense of threat), the three DSO symptom subscales (negative self-concept, affective dysregulation, disturbances in relationships), and the two items measuring dissociation. Analyses were performed with 2–8 cluster models to determine the best-fitting number of groups. Best fit was determined using the following criteria: 1) Bayesian information criterion (BIC), with lower values indicating better model fit; 2) Akaike information criterion (AIC), with lower values indicating better model fit; 3) entropy value, with higher values indicating better classification quality; 4) Lo-Mendell-Rubin-adjusted likelihood ratio test (which compares the fit of a given model versus the same model with one fewer profile); and 5) theoretically meaningful interpretation (fit statistics derived from Nylund-Gibson and Choi [42] and Weller et al. [59]). Models were estimated using a Maximum Likelihood Estimator (MLE) implemented via the Expectation–Maximization (EM) algorithm, with 20 iterations. Once a final model was selected, each participant was assigned a probability of profile membership and allocated to the profile with the highest probability. Exact subgroup sample sizes were not reported where *n* < 5 as per data privacy guidelines (NSW Ministry of Health, [41]).

Differences between profiles in ED symptoms and other continuous outcomes were tested using analysis of covariance (ANCOVA). Robust standard errors were calculated using the White (HC3) adjustment due to heteroscedasticity (R package *car* [21]). Where significant differences by trauma group were found, post-hoc pairwise comparisons were conducted with robust covariance matrices, using the Tukey adjustment for multiple comparisons (R package *emmeans* [31]). Due to count data with positively skewed distributions, differences between profiles in frequency of ED behaviours were analysed using negative binomial regression, with Bonferroni-adjusted pairwise comparisons conducted where the omnibus likelihood ratio test was significant. Associations between specific ED diagnoses and latent profile were examined using binomial logistic regression with likelihood ratio tests comparing models with and without the inclusion of latent profile. All tests of differences in outcome variables and ED presentation by profile were adjusted for level of care. Missing data were handled listwise.

## Results

Participants were predominantly women (91.8%) born in Australia (61.9%) or New Zealand (24.1%), with 3.70% of the sample identifying as Indigenous Australian, Māori, or Pacific Islander. Age ranged from 18 to 77 years, with a mean age of 27.46 years (*SE* = 0.51). Most of the sample were employed (32.8% full-time, 15.9% part-time) or students (33.3%).

The average age of ED onset was 16.17 years (*SE* = 0.34). Most of the sample (65.3%) had not required prior hospitalisation for their ED. The most common ED presentations were AN restrictive (38.5%) and binge-purge subtypes (17.8%), OSFED atypical AN (14.8%), BN (10.1%), and BED (7.7%).

Approximately half of participants scored above the severity threshold (at least one symptom from each subscale endorsed as ‘moderately’ or higher) for DSO symptoms (*n* = 171, 48.0%) and more than a quarter met the severity threshold for PTSD symptoms (*n* = 96, 27.0%). This resulted in probable diagnostic rates of 22.8% for CPTSD and 3.9% for PTSD (based on ICD-11 criteria).

### Latent profiles

Fit indices for the 2–8 cluster solutions are displayed in Table 1. Following consultation between co-authors SD, NT, DM and MG, who include experienced ED and trauma clinicians, the four-profile solution was chosen to represent the most parsimonious model based on model fit indices and ease of interpretation. Although more complex models had better fit indices, they were less theoretically meaningful and resulted in issues with small cell sizes. There were no issues with convergence.

**Table 1:** LPA model fit statistics

Profiles	ll	AIC	BIC	AICc	CAIC	Entropy	LMR	*p* > LMR
2	− 5447.84	10,939.69	11,025.30	10,942.67	11,047.30	0.9211		
3	− 5180.91	10,421.82	10,538.57	10,427.44	10,568.57	0.9232	522.78	< .001
4	− 5097.70	10,271.41	10,419.29	10,280.58	10,457.29	0.9201	162.95	< .001
5	− 5029.37	10,150.74	10,329.76	10,164.47	10,375.76	0.9144	133.82	.003
6	− 4991.32	10,090.64	10,300.78	10,109.98	10,354.78	0.9109	74.52	.016
7	− 4961.42	10,046.85	10,288.13	10,072.98	10,350.13	0.9206	58.55	.037
8	− 4942.31	10,024.63	10,297.04	10,058.79	10,367.04	0.8995	37.43	.447

*AIC *Akaike Information Criterion, *AICc *corrected Akaike Information Criterion, *BIC *Bayesian Information Criterion, *CAIC * Consistent Akaike Information Criterion,* LMR *Lo-Mendell-Rubin-adjusted likelihood ratio test statistic

The profiles emerging from the four-group solution were characterised as follows:

1. High DSO profile (with high DSO symptoms, low PTSD symptoms and moderate dissociation);
2. High CPTSD profile (with severe PTSD and severe DSO symptoms, and severe dissociation);
3. General distress profile (moderate symptoms on all indicator variables); and.
4. No/mild trauma-related symptoms profile (low on all indicator variables).

**Fig. 1:**
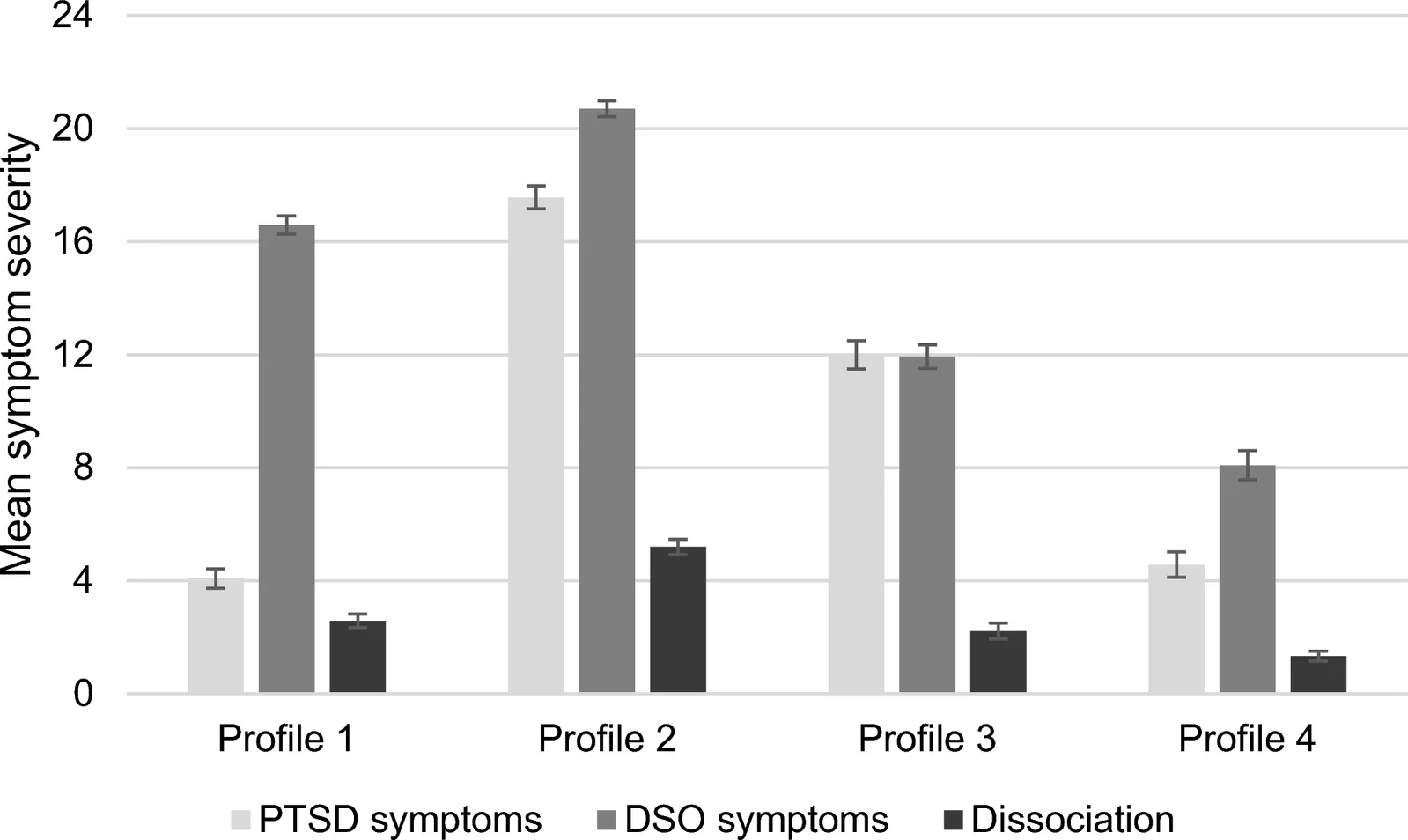
Average trauma-related symptoms by latent profile. *Note*. Error bars represent standard error. DSO = disturbances in self-organisation; PTSD = post-traumatic stress disorder

Prevalence and mean severity of trauma-related symptoms for each profile are reported in Tables 2 and 3. The pattern of trauma-related symptoms across groups is further depicted in Fig. 1.

**Table 2:** Prevalence of trauma-related profiles by level of care

	*n* (%) by profile				*n*
	1	2	3	4	
Total sample	93 (25.83%)	94 (26.11%)	46 (12.78%)	127 (35.28%)	360*
Residential	24 (34.29%)	37 (52.86%)	< 10 (~ 10.00%)	NR	70
Day program	5 (25.00%)	11 (55.00%)	NR	NR	20
Outpatient	64 (23.70%)	46 (17.04%)	35 (12.96%)	125 (46.30%)	270

*NR* Not reported due to small cell sizes. * Reflects missing data *n*=18 for trauma-related symptom measure

**Table 3:** Mean severity of trauma-related symptoms by latent profile

Trauma-related variable	Profile 1		Profile 2		Profile 3		Profile 4	
	Mean	SE	Mean	SE	Mean	SE	Mean	SE
PTSD symptoms (total)	4.08	0.34	17.57	0.41	12.00	0.50	4.57	0.45
Re-experiencing	0.76	0.10	5.11	0.19	2.74	0.24	0.46	0.07
Avoidance	1.71	0.18	6.17	0.16	4.48	0.25	0.81	0.10
Sense of current threat	1.60	0.18	6.30	0.19	4.80	0.31	0.74	0.10
DSO symptoms (total)	16.59	0.32	20.70	0.28	11.93	0.42	8.09	0.52
Affective dysregulation	4.89	0.19	6.47	0.14	4.57	0.21	1.34	0.10
Negative self-concept	6.07	0.17	7.28	0.12	3.80	0.31	1.16	0.12
Disturbances in relationships	5.61	0.15	6.97	0.14	3.57	0.24	0.96	0.09
Dissociation	2.58	0.24	5.20	0.27	2.22	0.28	1.33	0.18

*DSO* disturbances in self-organisation,* PTSD* post-traumatic stress disorder,* SE* standard error

Group membership was not significantly associated with gender (*χ*^*2*^ [6] = 2.47, *p* = 0.872) or age (*F*[3] = 1.00, *p* = 0.393), hence these demographic variables were not adjusted for in subsequent analyses. Profiles did significantly differ by level of care, *χ*^*2*^ (6) = 74.95, *p* < 0.001. In particular, higher levels of care saw greater proportional membership in group 2 (high CPTSD) and lower membership in group 4 (no/mild trauma-related symptoms).

A post-hoc power analysis indicated adequate power (0.99) for detecting differences between the four profile on subsequent analyses, at an alpha level of 0.05 and controlling for level of care.

### Latent profile associations with ED and other psychological outcomes

There was no significant association between trauma profile and any of the examined ED diagnoses (AN restrict and binge-purge subtypes, atypical AN, BN, and BED), adjusting for level of care (all *p*s > 0.05 for trauma profile, increase in Nagelkerke *R*^2^ = 0.01–0.08 indicating small effect size). Significant differences were found between profiles on all continuous ED and other psychological outcomes except frequency of objective binge episodes and baseline BMI. Effect sizes for outcomes with significant differences by profile were large for continuous outcome variables (η^2^_p_ > 0.14) and small to medium for significant differences in frequency of ED behaviours (φ = 0.12–0.41). Pairwise comparisons typically showed more severe ED and other psychological symptoms in group 2 (high CPTSD) than groups 3 (general distress) and 4 (no/mild trauma-related symptoms). Significant differences between group 1 (high DSO only) and group 2 (high CPTSD) were evident for global ED symptoms, eating concerns, purging episodes, and anxiety, such that group 2 showed more severe symptoms than group 1. Outcomes of these analyses are displayed in Table 4.

**Table 4:** Differences in eating disorder symptoms by latent profile, adjusted for level of care

Variable (continuous data)	Mean (SE) by profile				*F*	*p*	η^2^_p_	Significant pairwise comparisons
	1	2	3	4				
Global ED symptoms (EDE-Q)	4.22 (0.11)	4.75 (0.10)	3.53 (0.21)	3.36 (0.13)	13.06	< .001***	0.18	1 < 2 1 > 3 1 > 4 2 > 3 2 > 4
Dietary restraint	3.56 (0.17)	4.26 (0.16)	2.74 (0.28)	2.71 (0.16)	7.13	< .001***	0.14	2 > 3 2 > 4
Eating concerns	3.58 (0.12)	4.20 (0.12)	3.02 (0.21)	2.86 (0.13)	12.18	< .001***	0.15	1 < 2 1 > 4 2 > 3 2 > 4
Shape concerns	4.91 (0.11)	5.30 (0.10)	4.10 (0.24)	3.97 (0.15)	10.78	< .001***	0.15	1 > 3 1 > 4 2 > 3 2 > 4
Weight concerns	4.62 (0.14)	5.12 (0.12)	4.03 (0.24)	3.75 (0.15)	9.49	< .001***	0.13	1 > 4 2 > 3 2 > 4
Eating disorder-related impairment (CIA)	34.91 (1.04)	39.68 (0.93)	29.04 (1.41)	24.79 (0.99)	18.90	< .001***	0.29	1 > 3 1 > 4 2 > 3 2 > 4
Depression								
PHQ-9	16.79 (0.99)	23.02 (0.55)	15.82 (2.19)	10.00 (1.00)	33.07	< .001***	0.37	1 < 2 1 > 4 2 > 3 2 > 4
DASS-D	25.94 (1.18)	28.83 (1.27)	19.49 (1.53)	15.30 (0.89)	31.95	< .001***	0.26	1 > 3 1 > 4 2 > 3 2 > 4
Anxiety								
GAD-7	13.55 (0.98)	17.63 (0.56)	14.18 (1.32)	7.50 (3.50)	6.28	< .001***	0.22	1 < 2
DASS-A	13.63 (1.03)	22.17 (1.46)	13.37 (1.24)	9.84 (0.68)	19.96	< .001***	0.22	1 < 2 1 > 4 2 > 3 2 > 4
BMI	22.78 (0.79)	22.29 (0.84)	24.10 (1.38)	24.43 (0.66)	0.15	.932	0.02	n.s

Variable (count data)	Mean (SE) by profile				*LR χ*^*2*^	*p*	φ	Significant pairwise comparisons
	1	2	3	4				
Objective binges	6.77 (0.95)	6.16 (0.92)	6.20 (1.31)	6.65 (0.74)	4.57	.335	0.07	n.s
Subjective binges	6.45 (0.86)	9.38 (1.00)	4.61 (0.98)	6.49 (1.07)	14.87	.005**	0..12	2 > 3
Purging	6.59 (1.67)	10.37 (1.89)	3.46 (1.65)	2.42 (0.52)	180.81	< .001***	0.41	1 < 2 1 > 4 2 > 3 2 > 4
Compulsive exercise	7.83 (1.17)	12.28 (1.16)	7.60 (1.64)	6.97 (0.95)	29.58	< .001***	0.17	n.s

*BMI* body mass index (at treatment admission), * CIA *Clinical Impairment Assessment, * DASS-A, DASS-D* Depression Anxiety and Stress Scale (anxiety and depression subscales; measure of anxiety used for outpatient sample);* ED* eating disorder, * EDE-Q* Eating Disorder Examination Questionnaire,* GAD-7* Generalised Anxiety Disorder Screener-7 (measure of anxiety used for residential and day program samples),* n.s.* not significant at *p* > .05, *PHQ-9 *Patient Health Questionnaire (measure of depression used for residential and day program samples),* SE* standard error

*LR χ*^2^ likelihood-ratio chi-square,* φ* Cramer’s V effect size (0.1 = small, 0.3 = medium, 0.5 = large); η^2^_p_ = partial eta-squared (effect size; 0.01 = small, 0.06 = medium, 0.14 = large)

^*^ *p* < .05. ** *p* < .01. *** *p* < .001

## Discussion

The current study conducted the first latent profile analysis of trauma-related symptom profiles in individuals with EDs. Based on fit indices, support was found for a four-factor model consisting of a high DSO only profile, a CPTSD profile, a general distress profile, and a no/mild trauma-related symptoms profile. Dissociation appeared to co-vary with severity of DSO and PTSD symptoms, and was most severe in the CPTSD group where PTSD and DSO symptoms were greatest (CPTSD profile). Prevalence rates for the high DSO and CPTSD groups were each approximately 26%, aligning with previous research suggesting that over 50% of ED treatment-seekers report severe DSO symptoms but only half of these (one in four) meet diagnostic criteria for CPTSD (due to criteria requiring high levels of both PTSD and DSO symptoms) [11, 12].

Consistent with previous research on associations of severe PTSD and DSO symptoms and diagnosable CPTSD with elevated ED psychopathology [11, 12], the high DSO and CPTSD groups were each more severe than the general distress and no/mild trauma-related symptoms groups on total ED symptoms and ED-related impairment. Further significant differences between profiles were evident on ED symptom subscales, depression, anxiety, and frequency of subjective binge eating episodes, purging, and compulsive exercise, with the CPTSD group typically reporting more severe symptoms than the general distress and no/mild symptoms groups. The CPTSD group was significantly more severe than the high DSO group on global ED symptoms, eating concerns, purging, and anxiety, but not the other outcomes. This may suggest that severe DSO symptoms drive much of the association with ED psychopathology and other outcomes, but that the additional presence of high PTSD symptoms and high dissociation compound the risk of a severe ED presentation. These findings align with previous research implicating DSO symptoms in poorer mental wellbeing even in the absence of PTSD symptoms [18, 49, 54]. A corresponding severe PTSD symptoms-only profile was not found in the current study, which may be reflective of variation in diagnostic criteria between ICD-11 and DSM-5 classification systems as well as more frequent occurrence of CPTSD among ED populations [11, 12].

While the latent profiles found in the current study did significantly differ in severity of ED psychopathology, anxiety, and depression, the same was not found for frequency of objective binge episodes, ED diagnostic presentations, and BMI (after accounting for level of care). As significant differences *were* found for subjective binge episodes and purging, this presents mixed findings in relation to previous research suggesting strong links between trauma exposure or PTSD with binge eating, purging, and BN [22, 39, 57]. Partially conflicting findings in the current study may be due to low rates of BN and BED in the sample (< 20%), with AN subtypes more frequently represented (and significant differences between groups correspondingly found for purging and subjective binge episodes). Similarly, the high numbers of AN cases in the sample may have contributed to limited variation in BMI. Our finding that frequency of compulsive exercise differed by profile adds to very limited literature on potential associations between trauma and compulsive exercise behaviour [1]. As may be expected given previous findings of higher prevalence of PTSD and CPTSD in ED intensive care settings [6, 11, 12, 17], participants attending residential and day program services were more likely to be represented in the high DSO and CPTSD groups than the no/mild symptoms group, with the opposite found for outpatient treatment-seekers.

### Strengths, limitations, and future directions

This has been the first study to use latent profile analysis to explore patterns of trauma-related symptoms in individuals with EDs. By including symptoms from PTSD and CPTSD criteria as well as items exploring dissociation, our findings provide a more thorough representation of trauma-related presentations than studies that have focused on PTSD alone. Other strengths of the study included the use of participants across three levels of care (residential, day program, and outpatient), while controlling analyses for the effect of treatment setting.

Although the four-profile solution was supported by recognised fit criteria, interpretation of the latent profile analysis also relied on the greater theoretical meaningfulness and clinical utility of this solution. Replication in future studies will assist in verifying these findings and larger sample sizes may assist in uncovering less prevalent yet meaningful profiles (e.g., a high PTSD-only profile). As we only examined associations between the latent profile and ED symptoms at treatment admission, further research is also needed to determine whether these groups are predictive of variation in outcomes at discharge and follow-up. While the EDE-Q is a widely used measure of ED symptoms and its internal consistency was supported in the current sample, there is mixed evidence for the structural validity of its subscales [26]. A brief measure of dissociation was used to reduce participant burden but this would be improved in future research through the use of longer validated scales (for a review of measures, see Wainipitapong et al. [58]).

Although previous research supports PTSD, DSO, and dissociative symptoms as trauma-related phenomena [20, 28], we did not include a measure of lifetime trauma exposure and so are unable to validate whether participants in the high DSO and CPTSD profiles had experienced traumatic event/s. As such, future studies should consider including a measure of lifetime trauma exposure, including to determine whether specific traumatic events such as childhood sexual abuse are implicated in the trauma-related profiles observed. Exposure to childhood trauma is common among individuals with eating disorders [35] and specific types of childhood trauma (including childhood sexual abuse) and polyvictimisation have been shown to predict CPTSD symptomatology [24, 25]. Finally, samples that include greater numbers of individuals with BN and BED will assist in determining whether differences in profiles may be seen by ED diagnostic presentation.

### Implications

As the two profiles with high DSO symptoms (high DSO-only and CPTSD groups) were more common in individuals attending higher levels of ED care, this suggests the particular relevance of addressing these types of comorbid trauma presentations in intensive ED services. The greater need to address complex comorbidities in higher levels of care is at odds, however, with the specialised, ED-focused approach typically used in such settings, in contrast to the often more tailored and flexible approach more easily afforded in outpatient psychological treatment. This suggests a dilemma for intensive ED services: individuals attending such services do so because of the pressing need to address their ED, and the therapeutic approach is as such designed to reduce ED psychopathology as effectively and efficiently as possible. However, the more severe ED presentations seen in these settings are also more likely to co-occur with (and may in part be caused or maintained by) severe trauma-related symptoms, which are likely to go unaddressed given that most inpatient and residential ED care models do not include trauma-focused therapies and may not adhere to principles of trauma-informed care (see [5]).

The question thus becomes whether individuals with EDs would benefit from greater targeted use of trauma-focused therapies in higher levels of care (despite likely necessitating fewer resources directed towards the ED for these individuals), or whether trauma-focused treatment should be delayed until stepdown outpatient care. As trauma-related symptoms are theorised to maintain ED symptoms [55] and have been shown to predict slower improvement from ED residential care [13, 14], finding ways to integrate ED- and trauma-focused therapies across levels of care may help in improving treatment outcomes for the large number of individuals with some degree of comorbidity. Recent findings from Project Recover—a trial of integrated ED and PTSD treatment—supported a staged approach in which concurrent ED and PTSD therapies follow stabilisation of the ED [56]. Integrated use of trauma-focused therapies is likely to be most important for individuals with severe DSO symptoms (from either high DSO or CPTSD profiles), as there was no difference in ED symptoms between groups exhibiting moderate versus no/mild trauma-related symptoms (on both DSO and PTSD domains).

As DSO symptoms emerged as the defining feature of the two trauma profiles associated with more severe ED presentations in the current study, it is worth noting the ongoing debate as to the potential transdiagnostic relevance of DSO beyond trauma-related presentations. DSO have been shown to be present in other disorders such as depression and anxiety [15], but also demonstrate clear discriminant validity from disorders with similar symptoms (e.g., emotion dysregulation, relationship difficulties) such as borderline personality disorder [23]. The current state of evidence thus appears to support DSO as distinctly trauma-related in the sense of representing a reaction to experiences of interpersonal betrayal [19]. While there is very limited literature on the treatment implications for when DSO are present in the absence of PTSD symptoms, relevant approaches may include use of psychotherapies shown to be effective for DSO symptoms; for example, dialectical behaviour therapy (DBT) targets emotion dysregulation and there is emerging evidence that it can be adapted for CPTSD specifically [4]. Finally, the current findings suggest the utility of measures that can distinguish between PTSD and DSO symptoms (such as the ITQ, rather than measures based on DSM-5 PTSD criteria) in order to capture the complex trauma-related presentations that are most likely to be observed in ED populations.

## Conclusion

Trauma-related symptoms are common in individuals with EDs but understanding their association with ED psychopathology requires going beyond standard diagnostic criteria. The current study indicates that severe DSO symptoms—in the presence and absence of severe PTSD symptoms and dissociation—create two distinct comorbid trauma-related presentations in EDs, both of which are associated with more severe ED symptoms, anxiety, and depression. The combination of high DSO, PTSD, and dissociative symptoms appears to be a marker of a particularly severe ED subgroup. Associations between trauma-related subgroups and outcomes were evident across attitudinal ED symptoms, ED behaviours, and mood/anxiety. Further research is needed to replicate these latent profiles in ED populations to determine whether such profiles can be used to assist with treatment planning in ED services, such as to guide decision-making around the incorporation of trauma-focused therapies.

## Data Availability

The datasets generated and/or analysed during the current study are not publicly available due to privacy/ethics restrictions but are available from the corresponding author on reasonable request.
